# Supplementary material for: Investigating Vα7.2+/CD161− T Cell and MAIT Cell Profiles Using Flow Cytometry in Healthy Subjects and Subjects with Atopic Dermatitis
Source: Int J Mol Sci. 2024 Mar 20;25(6):3486. doi: 10.3390/ijms25063486 (PMC10970526; doi:10.3390/ijms25063486)
Supplement: Supplementary file 1 [file ijms-25-03486-s001.zip › ijms-2897601-supplementary.pptx]

## Slide 1
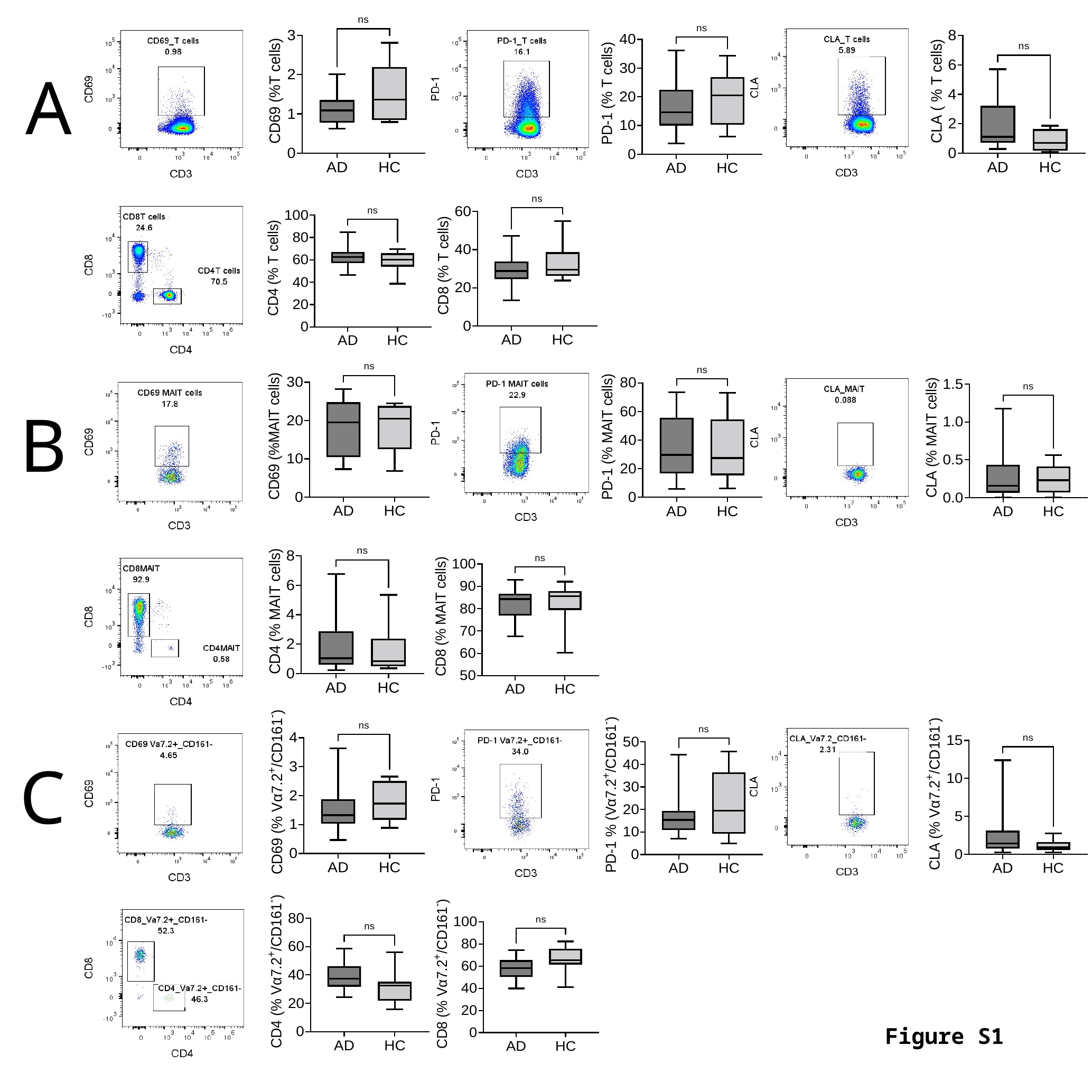

A
B
C
Figure S1

## Slide 2
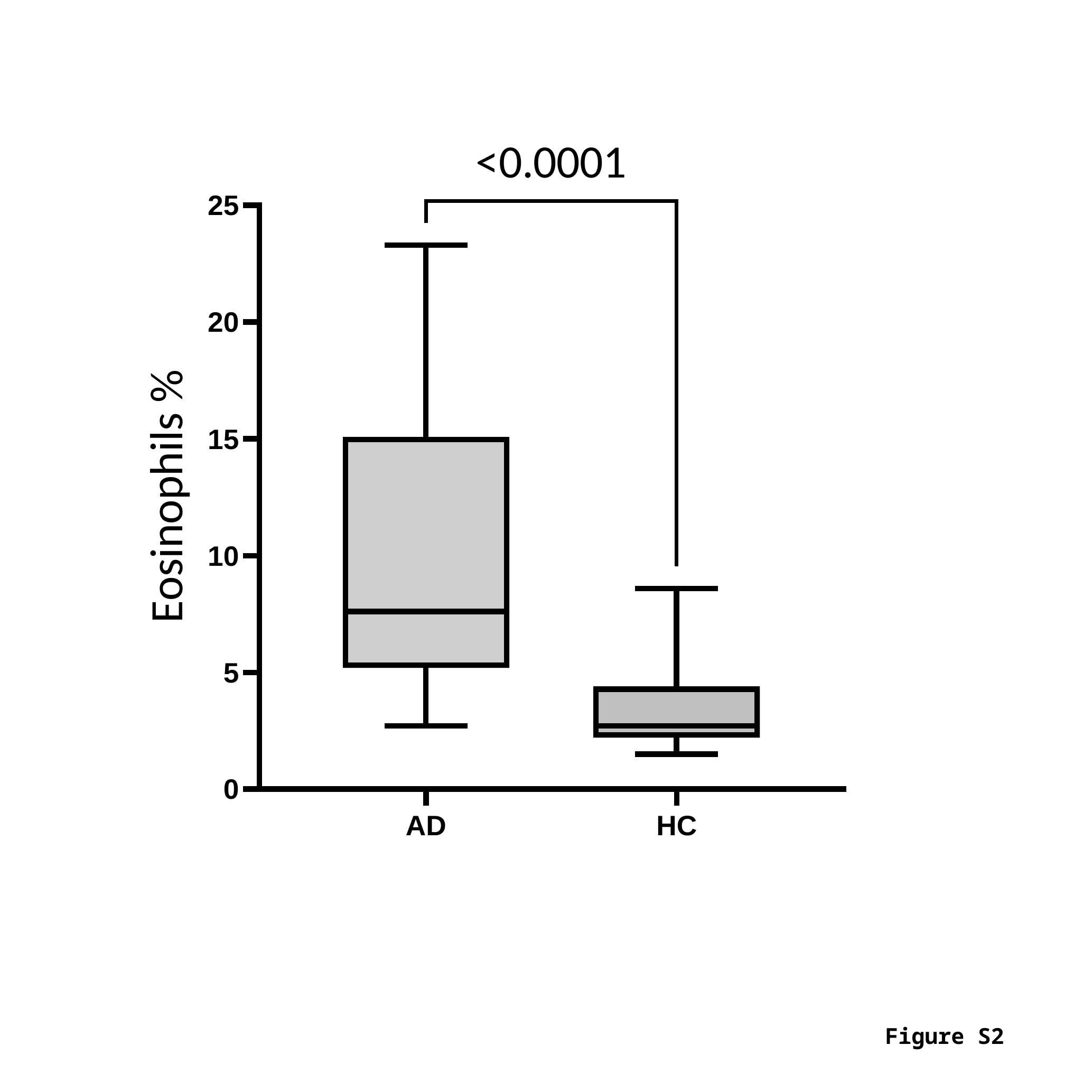

Figure S2

## Slide 3
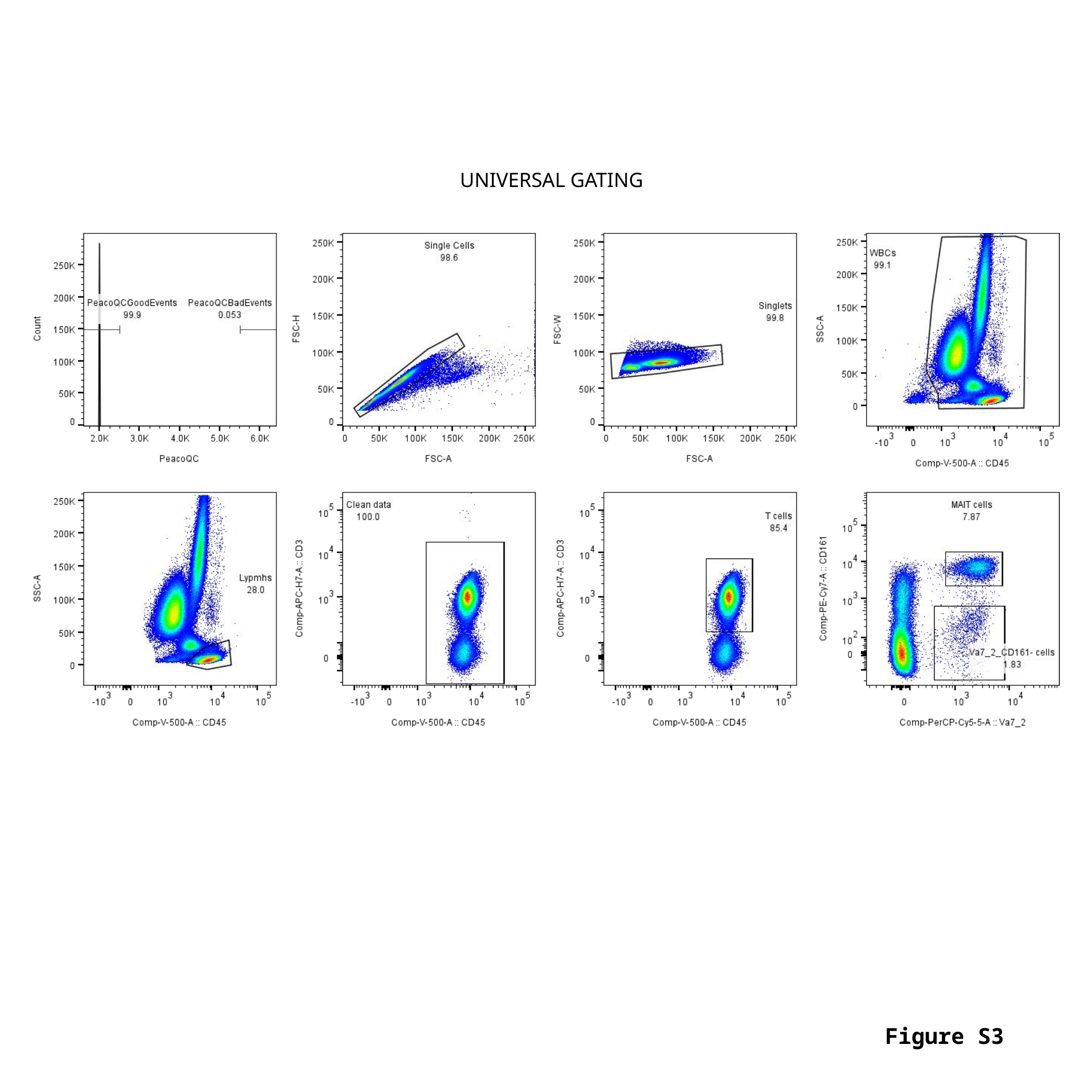

UNIVERSAL GATING
Figure S3

## Slide 4
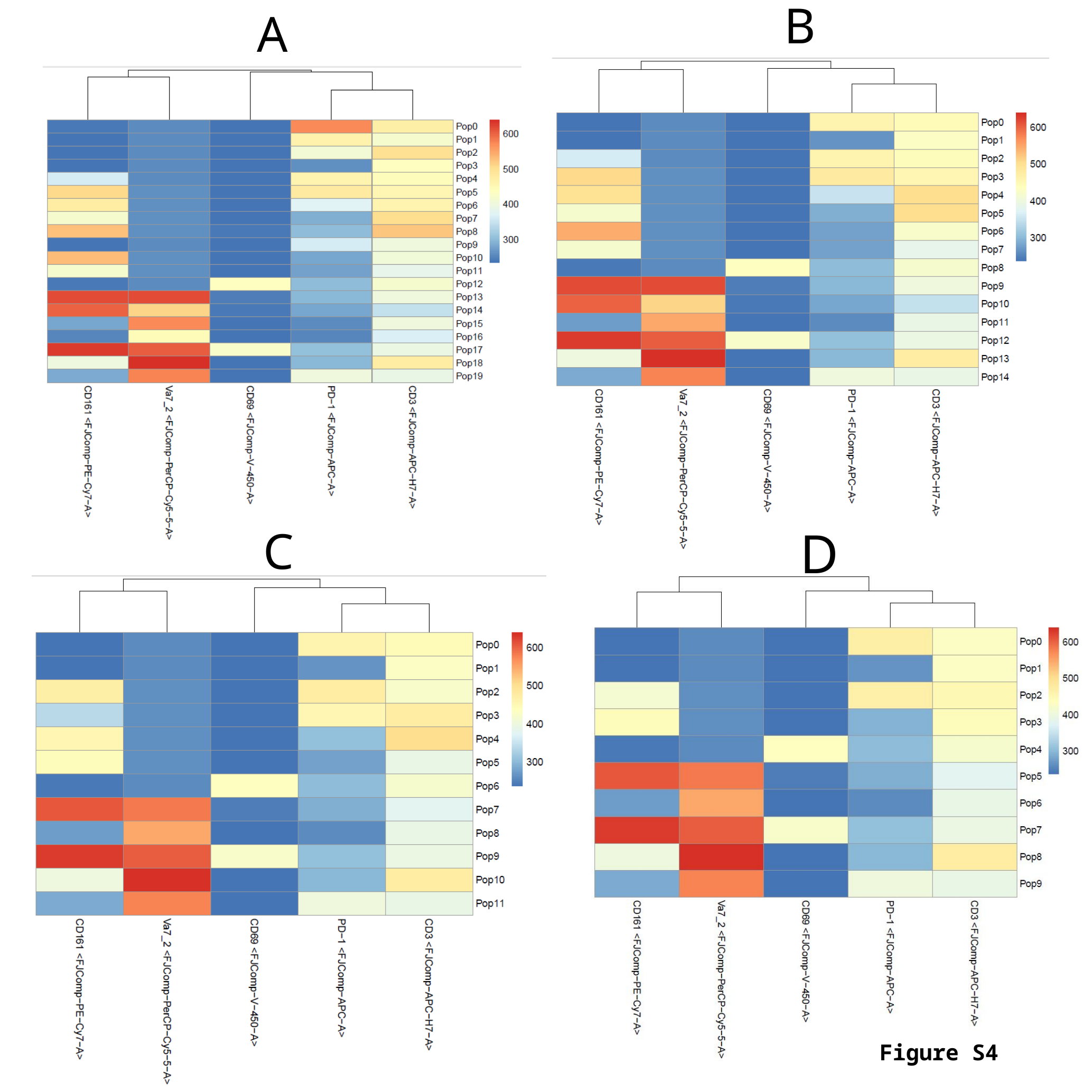

A
B
C
D
Figure S4

## Slide 5
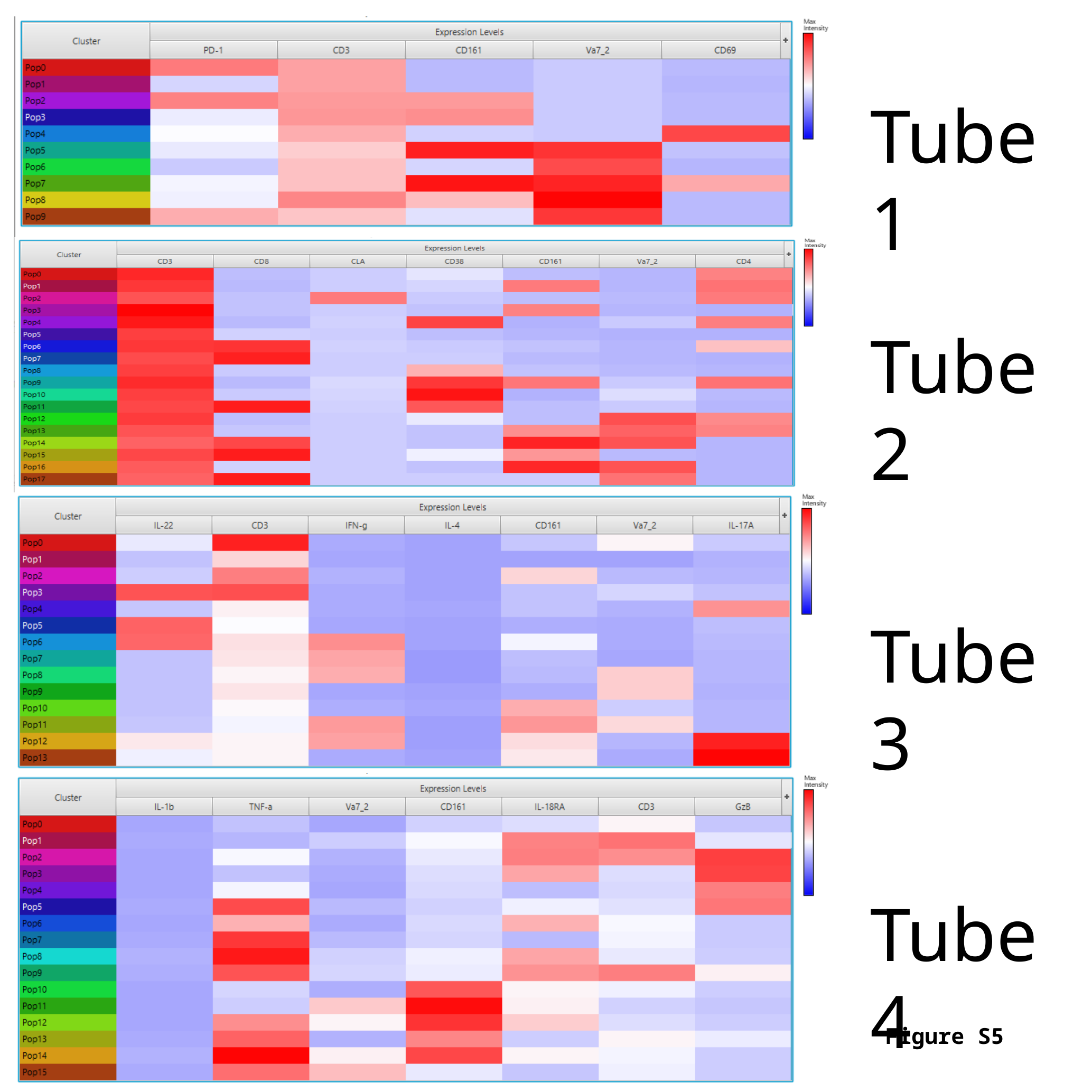

Tube 1
Tube 2
Tube 3
Tube 4
Figure S5

## Slide 6
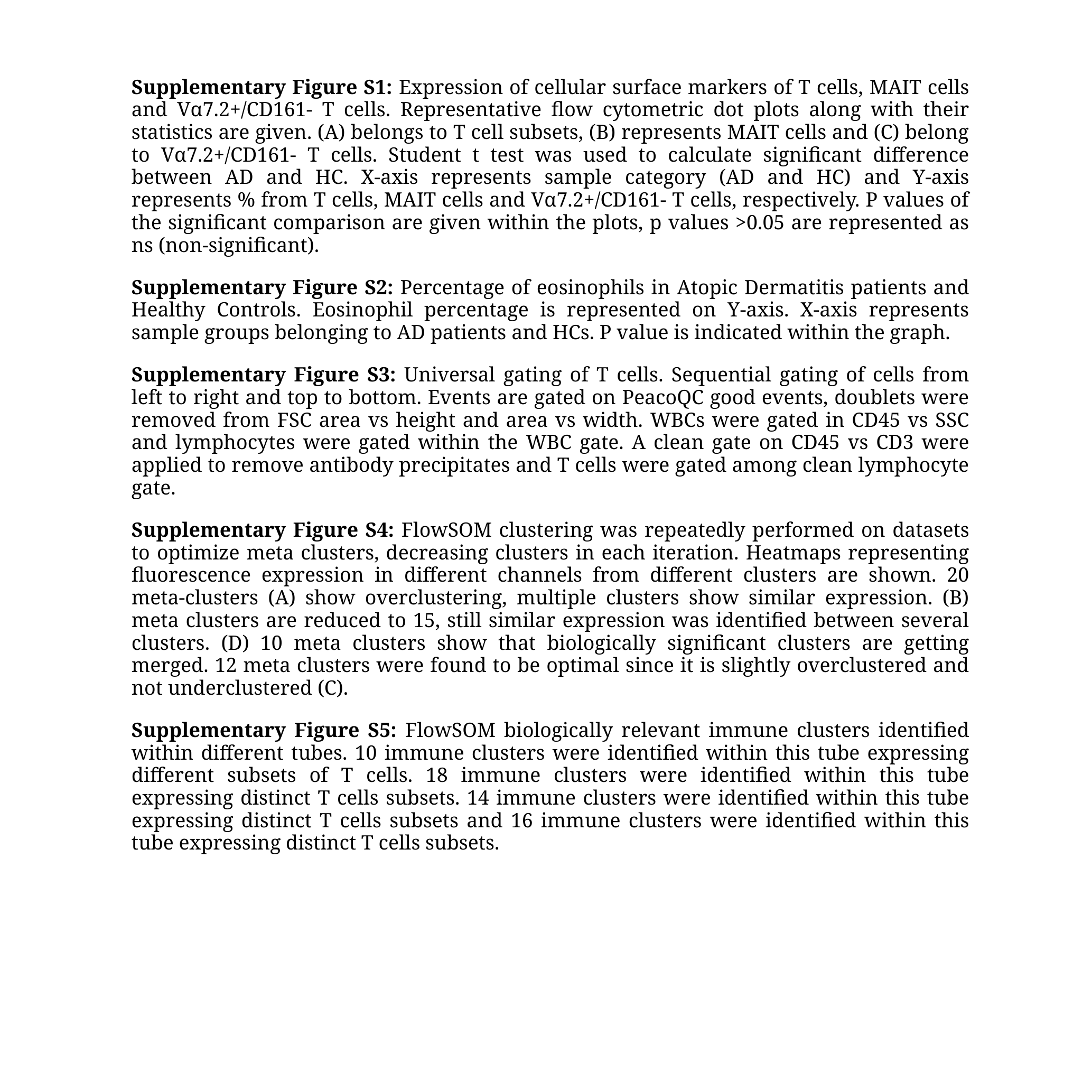

Supplementary Figure S1: Expression of cellular surface markers of T cells, MAIT cells and Vα7.2+/CD161- T cells. Representative flow cytometric dot plots along with their statistics are given. (A) belongs to T cell subsets, (B) represents MAIT cells and (C) belong to Vα7.2+/CD161- T cells. Student t test was used to calculate significant difference between AD and HC. X-axis represents sample category (AD and HC) and Y-axis represents % from T cells, MAIT cells and Vα7.2+/CD161- T cells, respectively. P values of the significant comparison are given within the plots, p values >0.05 are represented as ns (non-significant).
Supplementary Figure S2: Percentage of eosinophils in Atopic Dermatitis patients and Healthy Controls. Eosinophil percentage is represented on Y-axis. X-axis represents sample groups belonging to AD patients and HCs. P value is indicated within the graph.
Supplementary Figure S3: Universal gating of T cells. Sequential gating of cells from left to right and top to bottom. Events are gated on PeacoQC good events, doublets were removed from FSC area vs height and area vs width. WBCs were gated in CD45 vs SSC and lymphocytes were gated within the WBC gate. A clean gate on CD45 vs CD3 were applied to remove antibody precipitates and T cells were gated among clean lymphocyte gate.
Supplementary Figure S4: FlowSOM clustering was repeatedly performed on datasets to optimize meta clusters, decreasing clusters in each iteration. Heatmaps representing fluorescence expression in different channels from different clusters are shown. 20 meta-clusters (A) show overclustering, multiple clusters show similar expression. (B) meta clusters are reduced to 15, still similar expression was identified between several clusters. (D) 10 meta clusters show that biologically significant clusters are getting merged. 12 meta clusters were found to be optimal since it is slightly overclustered and not underclustered (C).
Supplementary Figure S5: FlowSOM biologically relevant immune clusters identified within different tubes. 10 immune clusters were identified within this tube expressing different subsets of T cells. 18 immune clusters were identified within this tube expressing distinct T cells subsets. 14 immune clusters were identified within this tube expressing distinct T cells subsets and 16 immune clusters were identified within this tube expressing distinct T cells subsets.
